# A phase 3, open-label, multicenter study of a 6-month pre-mixed depot formulation of leuprolide mesylate in advanced prostate cancer patients

**DOI:** 10.1007/s00345-019-02741-7

**Published:** 2019-04-03

**Authors:** Neal Shore, Ivan Mincik, Mark DeGuenther, Vladimir Student, Mindaugas Jievaltas, Jitka Patockova, Kelle Simpson, Chu-Hsuan Hu, Shih-Tsung Huang, Yuhua Li, Yisheng Lee, Ben Chien, John Mao

**Affiliations:** 1 Carolina Urologic Research Center, Suite B, 823 82nd Parkway, Myrtle Beach, SC 29579 USA; 2 Urocentrum Milab, s.r.o. Hollého 14/D, 080 01 Presov, Slovak Republic; 3 Urology Center of Alabama, 3485 Independence Drive, Homewood, AL 35209 USA; 4 Fakultní Nemocnice Olomouc Urologická Klinika, I. P. Pavlova 6, Olomouc, 779 00 Czech Republic; 5 Hospital of Lithuanian University of Health Sciences Kauno Klinikos, Eivenių 2, Kaunas, 50009 Lithuania; 6 QPS Holdings, LLC, Three Innovation Way, Suite 240, Newark, DE 19711 USA; 7 Department of Surgery and Urology, Chang Gung Memorial Hospital-Linkou, Chung Gung University, College of Medicine, Taoyuan, Taiwan; 8 Foresee Pharmaceuticals Co., Ltd., 3 F, No 19-3, Sanchong Road, NanKang Dist., Taipei, 11501 Taiwan, R.O.C.

**Keywords:** Prostate cancer, Leuprolide acetate, Luteinizing hormone–releasing hormone agonist, Testosterone, PSA, Androgen deprivation therapy

## Abstract

**Objectives:**

To determine the safety, efficacy and pharmacokinetic (PK) profile of a pre-mixed depot formulation of leuprolide mesylate subcutaneous injectable suspension (LMIS) 50 mg for up to 1 year of treatment for subjects with advanced prostate cancer.

**Patients and methods:**

In this open-label, multicenter study, prostate cancer patients with indication for androgen ablation therapy received two subcutaneous injection of LMIS 50 mg 6 months apart and were followed for an additional 6 months. Two efficacy primary end points were the percentage of subjects with a serum testosterone level ≤ 50 ng/dL by Day 28 as well as the percentage of subjects with similar testosterone suppression from Day 28 to Day 336.

**Results:**

Of the 137 enrolled subjects, 15 (10.9%) subjects did not complete the study, including 5 subjects who terminated early due to an adverse event. By Day 28, 98.5% (95% confidence interval 94.8–99.8) of the subjects achieved a castrate testosterone level. At the end of the study, 97% and 95.9% of the subjects had serum testosterone level ≤ 50 ng/dL and ≤ 20 ng/dL, respectively. LMIS 50 mg significantly reduced serum prostate-specific antigen levels after its first injection and this PSA declination effect remained until the end of the study. No statistically significant change was observed in worsening bone pain or urinary symptom assessments during the study. Hot flush (48.9%) and hypertension (14.6%) were the two most common adverse events reported.

**Conclusions:**

LMIS 50 mg, administered at 6-month intervals, effectively suppressed serum testosterone level, and demonstrated a consistent safety profile.

## Introduction

In 2018, the American Cancer Society estimates that prostate cancer is the most frequently diagnosed cancer and the second cause of cancer-related death for men in the US [1]. In the majority of these cases, the growth, proliferation and survival of prostate cancer cells are dependent upon androgen stimulation, predominantly via testosterone and dihydrotestosterone, produced within the Leydig cells of the testes [2]. Since the benefits of surgical castration on the outcomes of metastatic prostate cancer were first described in 1941 [3], androgen ablation remains the mainstay of treatment for men with advanced prostate cancer [4].

Pharmacologic LHRH drug development over the years demonstrated that chronic administration of synthetic long-acting analogs of the luteinizing hormone–releasing hormone (LH–RH) resulted in suppression of Leydig cell function, leading to the first use of these agents in the treatment of prostate cancer [5]. LH–RH analogs long-term administration eventually reduces the number of LH–RH receptors and inhibits gonadotropin synthesis and secretion [6, 7]. Within weeks of treatment, prostate cancer patients receiving LH–RH analogs exhibit suppression of serum testosterone level, similar to surgical castration [8]. Prostate cell division and growth are dependent on testosterone, and less testosterone results in more apoptotic cells in prostate; this is most important in the systemic treatment of prostate cancer.

Over the following decades, androgen ablation with LH–RH analogs has proven its clinical equivalence to bilateral orchiectomy [9], with the potential advantages of avoiding the irreversibility and adverse psychological effects of surgical castration [10] and also potentially allowing the intermittent administration of androgen suppression, if clinically indicated [11–13]. Several different LH–RH analogs were developed, studied and approved for the treatment of prostate cancer, including leuprolide, triptorelin, goserelin, histrelin and buserelin. These drugs may be administered as 1-, 3-, 4- or 6-month intramuscular or subcutaneous injections, or as a 12-month subcutaneous implant [14].

More recently, the leuprolide mesylate injectable suspension (LMIS) was developed as a pre-mixed drug product containing 50 mg leuprolide mesylate (equivalent to 42 mg of leuprolide free base) formulated in a solution of *N*-methyl-2-pyrrolidone and poly (d,l-lactide) to control and sustain the release of leuprolide over a 6-month period after subcutaneous administration. In addition to the convenience of clinical use, LMIS 50 mg was developed as a single, sterile, pre-filled syringe which is ready to use and does not require premixing prior to subcutaneous injection, thus eliminating a potential source of error which has been shown to affect the clinical efficacy of drug administration [15].

The present study was designed to determine the safety, tolerability, and efficacy of LMIS 50 mg for up to 1 year of exposure in subjects with advanced prostate carcinoma, as well as to evaluate the pharmacokinetic (PK) behavior of 50 mg LMIS following two separate subcutaneous injections given 6 months apart.

## Patients and methods

### Study design and patients

This was a multi-center, open-label, single-arm study conducted to assess the safety, efficacy and PK behavior of LMIS 50 mg in subjects with prostate cancer.

Eligible patients were age ≥ 18 years, had histologically confirmed prostate carcinoma, baseline morning serum testosterone level > 150 ng/dL, Eastern Cooperative Oncology Group (ECOG) performance status ≤ 2, adequate organ function, and were assessed by the physician investigator to be candidates for androgen ablation therapy. Patients who failed primary treatment (surgery/radiation) are still eligible.

Key exclusion criteria included concomitant use of chemotherapy, immunotherapy, cryotherapy, radiotherapy, or anti-androgen therapy, or within the previous 8 weeks, for treatment of carcinoma of the prostate; utilization of any LH–RH suppressive therapy within the previous 6 months; history of anaphylaxis or contraindication to any LH–RH analogs; clinical evidence of or risk for urinary obstruction; clinically significant abnormal electrocardiogram (ECG) and/or history of clinically significant cardiovascular disease; use of 5-alpha reductase inhibitor within the last 6 months; history or the presence of insulin-dependent diabetes mellitus (type I; the presence of well-controlled diabetes mellitus type II was allowed if only oral hypoglycemic was required); and use of systemic corticosteroids at a dose > 10 mg/day.

Part I was conducted to establish the safety of LMIS 50 mg with more frequent visits for safety monitoring. Patients received a single subcutaneous injection of LMIS 50 mg every 6 months (over the course of 2 doses on Day 1 and Day 168). The first 10 (of the 33) subjects served as a “sentinel” group for safety purposes and the Independent Data Monitoring Committee conducted interim safety reviews for these subjects at the end of Week 2, Month 1, Month 3, and Month 6. These four interim safety reviews were performed in addition to the interim review of safety data, serum leuprolide concentrations, and serum testosterone suppression status scheduled for the first 30 subjects following their Day 28 assessments in Part I.

If  ≥ 90% of Part I subjects (i.e., ≥ 27 of the 30 subjects) achieved suppression of serum testosterone to castrated levels within 1 month of the initial dose and demonstrated acceptable safety and tolerability, Part II of the study was opened to enroll approximately 100 subjects. After the second administration of LMIS 50 mg, all subjects were followed for safety, tolerability, efficacy, PK, and ancillary clinical and laboratory markers for an additional 6 months.

### Outcomes

The primary efficacy end points were the percentage of subjects with a serum testosterone ≤ 50 ng/dL (castration level) by Day 28 ± 1 (i.e., within 28 days following the first injection of investigational product) and the percentage of subjects with testosterone suppression (≤ 50 ng/dL) from Day 28 through Day 336. Insufficient suppression of testosterone was defined as suppression that did not occur by Day 28 or the occurrence of a testosterone level > 50 ng/dL between Day 28 and Day 336.

The efficacy of two separate doses of LMIS 50 mg was further analyzed by examining the percentage of subjects with the serum testosterone ≤ 20 ng/dL on Day 28 and Day 336.

Secondary end points included the proportion of subjects exhibiting post-suppression excursion of serum testosterone to > 50 ng/dL, either through “breakthrough” (i.e., episodes unrelated to LMIS 50 mg dosing), or through the “acute-on-chronic” effect (i.e., related to the second dose of LMIS 50 mg), which is commonly referred to as “surge”.

Secondary end points also included the effect of LMIS 50 mg on serum prostate-specific antigen (PSA) levels, serum LH levels, laboratory parameters, local skin tolerability, bone pain and urinary symptom exacerbations. The PK behavior of leuprolide was evaluated by full PK profiles from serum leuprolide concentrations in Part I subjects. Additional serum leuprolide concentration data were collected during Part II.

### Assessments

Pharmacokinetic (PK) and pharmacodynamics (PD) assessments of serum leuprolide concentrations and serum testosterone concentrations using a validated LC/MS/MS bioanalytical methods were performed at the bioanalytical laboratory. The PK parameters, including *C*_max_, *T*_max_, *C*_wk4_, *C*_mon6_, AUC_0–4wk_, AUC_0–6mon_ and *C*_avg_ (0–6 months), were determined using non-compartmental approaches using Phoenix WinNonlin^®^ (Certara USA, Inc., Princeton, NJ, USA).

The safety and tolerability of LMIS 50 mg were evaluated by analyzing adverse events by changes in: bone pain; urinary symptoms measurements by the AUA symptom score and by the Visual Analog Scale (VAS) scale; vital signs; physical examinations; laboratory tests; and 12-lead resting ECGs. Additional assessments were performed for local skin tolerability.

### Statistical analysis

The intention-to-treat (ITT) population was defined as any subject who received at least one dose of LMIS 50 mg and the per protocol (PP) population was defined as any subject who received two doses of LMIS 50 mg, followed the inclusion/exclusion requirements of the protocol, and had no major protocol violation. The ITT and PP populations were used in the efficacy analyses. Any subject receiving a dose of LMIS 50 mg was included in the safety analysis.

For primary efficacy end points, the percentage of subjects with a serum testosterone ≤ 50 ng/dL (castrate level) by Day 28 ± 1(day) was analyzed using a standard large sample approximation to a Binomial distribution. The percentage of subjects with testosterone suppression (≤ 50 ng/dL) from Day 28 to Day 336 was analyzed using a Kaplan–Meier approach, in which an event was defined as the occurrence in any subject with a serum testosterone level > 50 ng/dL at or before Day 336. The duration of time to an event was summarized by Kaplan–Meier plot, and presented as event number, percentage, 95% confidence interval (CI) for percentage, mean, median and 95% CI for median.

For descriptive statistics, continuous variables were presented as number of observations, mean, standard deviation, median, range, Hodges–Lehmann estimator and 95% CI. Categorical variables were presented by frequency and percentage. Changes from baseline were tested by a paired *t* test or Wilcoxon signed-rank test for continuous variables using a significance level of 0.05.

## Results

**Fig. 1:**
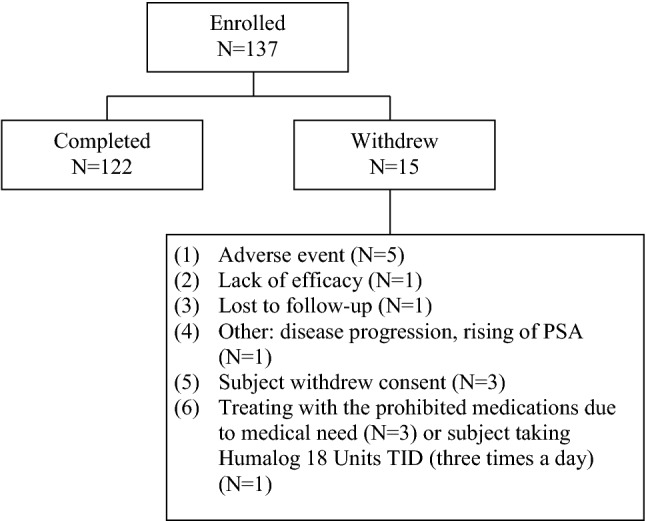
Disposition of patients

A total of 137 subjects were enrolled in this study, including 33 subjects in Part I and 104 subjects in Part II. Of the 137 enrolled subjects, 15 (10.9%) subjects did not complete the study, including 5 subjects who terminated early due to adverse event (Fig. 1). The median age of the entire cohort was 71.0 years and the median duration with diagnosed prostate carcinoma was 633 days. Almost 90% of enrolled subjects were Caucasian. Other baseline characteristics are detailed in Table 1.

**Table 1:** Baseline characteristics

Variable	Part I (*N* = 33)	Part II (*N* = 104)	Total (*N* = 137)
Age (years)			
Median (range)	74.0 (54–86)	70.0 (51–88)	71.0 (51–88)
Race			
White	25 (75.8%)	98 (94.2%)	123 (89.8%)
Black	4 (12.1%)	4 (3.8%)	8 (5.8%)
Asian	4 (12.1%)	1 (1.0%)	5 (3.6%)
Unknown	0 (0.0%)	1 (1.0%)	1 (0.7%)
Diagnosis (days)			
Median (range)	2254.0 (12–9066)	158.5 (0–13,290)	633.0 (0–13,290)
Staging			
I	1 (3.0%)	3 (2.9%)	4 (2.9%)
II	8 (24.2%)	23 (22.1%)	31 (22.6%)
III	5 (15.2%)	32 (30.8%)	37 (27.0%)
IV	9 (27.3%)	23 (22.1%)	32 (23.4%)
Unknown	10 (30.3%)	23 (22.1%)	33 (24.1%)
ECOG performance status			
0	30 (90.9%)	84 (80.8%)	114 (83.2%)
1	3 (9.1%)	19 (18.3%)	22 (16.1%)
2	0 (0.0%)	1 (1.0%)	1 (0.7%)

### Primary efficacy end points

The percentage of subjects with a serum testosterone ≤ 50 ng/dL (castrate level) by Day 28 was 98.5% (95% CI, 94.8–99.8) in the ITT population and 99.2% (95% CI 95.6–100.0) in the PP population, respectively.

**Fig. 2:**
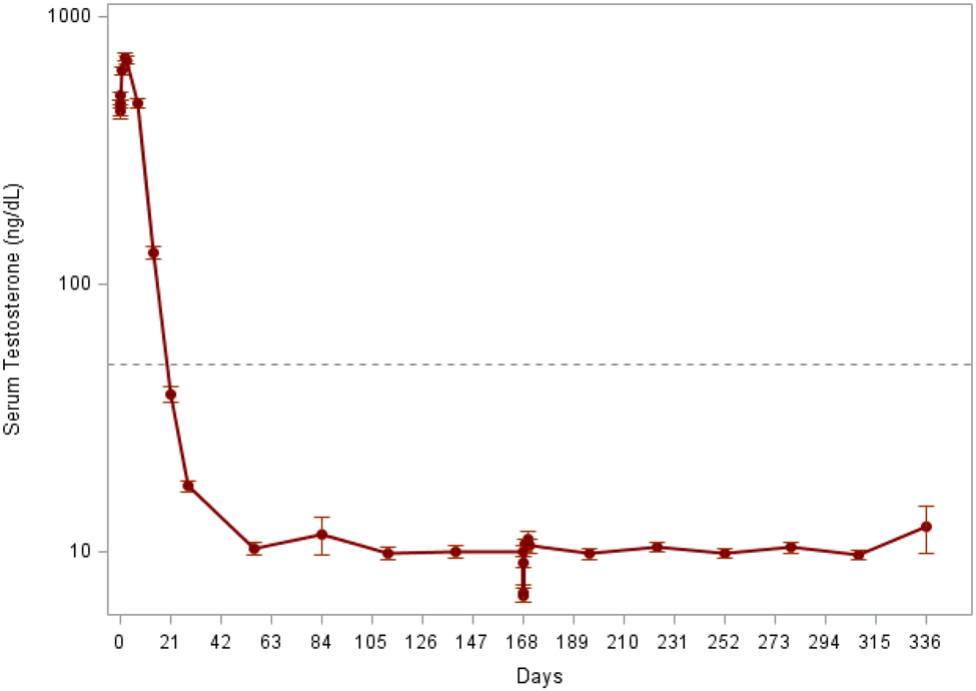
Mean serum testosterone over time in the ITT population

The percentage of subjects with testosterone suppression (≤ 50 ng/dL) from Day 28 to Day 336 was 97.0% (95% CI 92.2–98.9) in the ITT population and 97.6% (95% CI 92.7–99.2) in the PP population, respectively. The mean serum testosterone was below ≤ 50 ng/dL at 38.96 ± 30.18 ng/dL at Day 21 and remained below castration levels at 12.38 ± 28.21 ng/dL at Day 336 in the ITT population (Fig. 2).

Of 135 subjects who achieved the castrated level serum testosterone (≤ 50 ng/dL), 95 subjects (95/135, 70.4%) achieved serum testosterone level < 20 ng/dL on Day 28. On Day 336, all subjects who completed the study (122/122) achieved castrated serum testosterone level (≤ 50 ng/dL), among which 117 subjects (117/122, 95.9%) achieved serum testosterone level < 20 ng/dL.

### Secondary efficacy end points

Only two subjects (2/137, 1.5%) exhibited post-suppression elevations of serum testosterone to > 50 ng/dL after reaching castrate level of testosterone on Day 28. One was on Days 169–171, and the other was on Day 170. These transient elevations of serum testosterone levels were observed following the second administration of LMIS 50 mg on Day 168 and possibly related to the acute-on-chronic effect of LH. Both subjects had their serum testosterone levels below the castrate levels by the end of the study (Day 336). Besides these episodes, no other “breakthrough” episode of serum testosterone to > 50 ng/dL was observed in this study.

The administration of LMIS 50 mg significantly reduced the serum PSA levels after its first injection and this effect remained until the end of the study. In a post hoc analysis of the 99 subjects who had elevated PSA (> 4 ng/mL) at baseline, more than 90% subjects experienced a decrease in PSA levels on Day 28. The percentages of subjects with elevated PSA levels at baseline decreased by LMIS 50 mg injection in different visits are summarized in Table 2.

**Table 2:** Summary of PSA levels over time for subjects with elevated PSA at baseline (ITT population)

Visit	ITT population (*N* = 99)							
	Number of patients (percent)							
	*n*	< 50% decrease	50 to < 90% decrease	90 to < 95% decrease	≥ 95% decrease	Increase	≤ 4 ng/mL	> 4 ng/mL
Day 28	99	26 (26.3%)	65 (65.7%)	4 (4.0%)	1 (1.0%)	3 (3.0%)	47 (47.5%)	52 (52.5%)
Day 84	98	4 (4.1%)	31 (31.6%)	22 (22.4%)	41 (41.8%)	0 (0.0%)	75 (76.5%)	23 (23.5%)
Day 168	94	2 (2.1%)	24 (25.5%)	11 (11.7%)	57 (60.6%)	0 (0.0%)	80 (85.1%)	14 (14.9%)
Day 252	90	2 (2.2%)	16 (17.8%)	13 (14.4%)	58 (64.4%)	1 (1.1%)	76 (84.4%)	14 (15.6%)
Day 336	88	4 (4.5%)	17 (19.3%)	11 (12.5%)	55 (62.5%)	1 (1.1%)	76 (86.4%)	12 (13.6%)

**Fig. 3:**
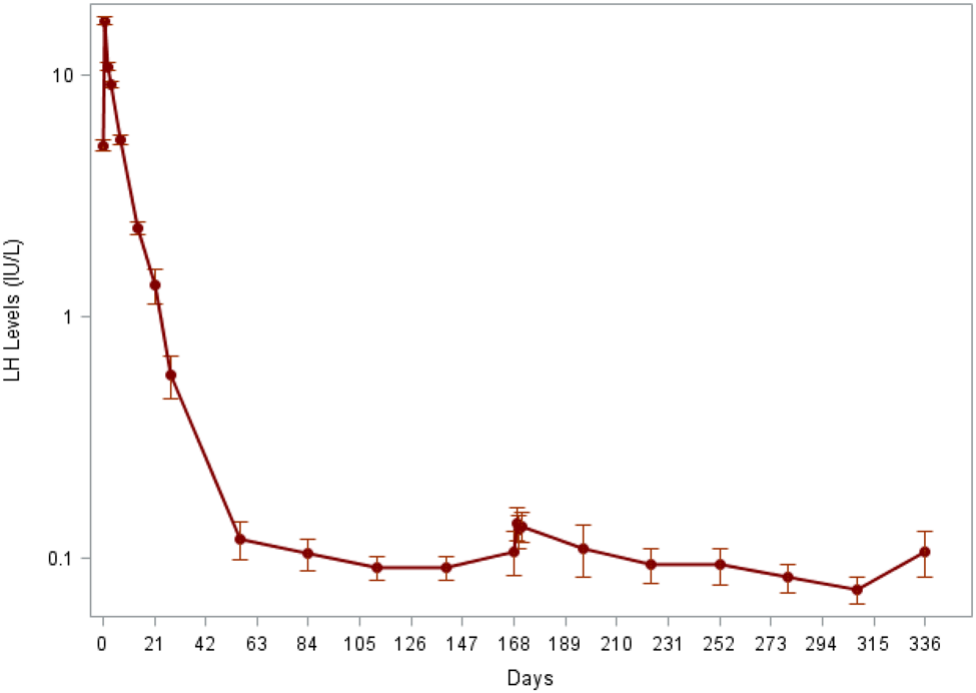
Mean LH levels over time from the local laboratory in the ITT population

In the ITT population, the mean LH level at baseline was 5.13 ± 3.04 IU/L and, as expected, an acute increase of mean serum LH level on Day 1 was observed after the first administration of LMIS 50 mg. A rapid decrease of serum LH levels was observed on Days 2 and 3 until it approached a plateau on Day 140. A slight increase in mean LH level was observed at the end of the study on Day 336 (Fig. 3).

### Safety

A total of 553 AEs from 114 subjects (114/137, 83.2%) were reported with at least one treatment-emergent adverse events (TEAEs) occurrence in this study (Table 3). The most common (≥ 5%) TEAEs observed was hot flush (48.9%), followed by hypertension (14.6%), pain in extremity (9.5%), injection site pain (7.3%), arthralgia (6.6%), fatigue (6.6%), nocturia (5.8%), back pain (5.1%) and nasopharyngitis (5.1%). With regard to the severity of TEAEs, most were mild or moderate.

**Table 3:** Summary of AEs (safety population)

Variables\status	Part I (*N* = 33)			Part II (*N* = 104)			Total (*N* = 137)		
	Event	Subject	(%)	Event	Subject	(%)	Event	Subject	(%)
TEAEs									
Total	160	31	93.9	393	83	79.8	553	114	83.2
TEAEs by severity									
Mild	116	29	87.9	279	76	73.1	395	105	76.6
Moderate	36	19	57.6	94	40	38.5	130	59	43.1
Severe	6	5	15.2	18	13	12.5	24	18	13.1
Hot flush	18	18	54.5	51	49	47.1	69	67	48.9
Hypertension	2	2	6.1	21	18	17.3	23	20	14.6
Pain in extremity	3	2	6.1	15	11	10.6	18	13	9.5
Injection site pain	6	5	15.2	7	5	4.8	13	10	7.3
Arthralgia	5	4	12.1	6	5	4.8	11	9	6.6
Fatigue	3	3	9.1	7	6	5.8	10	6	6.6
Nocturia	1	1	3.0	8	7	6.7	9	8	5.8
Back pain	4	4	12.1	3	3	2.9	7	7	5.1
Drug-related AEs									
Total	44	21	63.6	100	64	61.5	144	85	62.0
Drug-related AEs by severity									
Mild	35	19	57.6	81	56	53.8	116	75	54.7
Moderate	7	5	15.2	18	13	12.5	25	18	13.1
Severe	2	2	6.1	1	1	1.0	3	3	2.2
Hot flush	18	18	54.5	50	48	46.2	68	66	48.2
Injection site pain	6	5	15.2	7	5	4.8	13	10	7.3
Fatigue	2	2	6.1	6	6	5.8	8	8	5.8

Of 137 enrolled subjects, 62% subjects (85/137) were reported with drug-related AEs determined by investigators. The most common (≥ 5%) drug-related AEs observed were hot flush (48.2%), followed by injection site pain (7.3%) and fatigue (5.8%).

Five out of 137 subjects (3.6%) who experienced an adverse event (AE) discontinued from the study prematurely. These AEs included: acute kidney injury, atrial fibrillation, cerebrovascular accident, death, hormone-refractory prostate cancer, and prostate cancer metastatic. None of these AEs were determined to be related to the administration of 50 mg LMIS.

Of 34 serious AEs, only three were determined as related to the investigational product by the primary investigators, and agreed by the chief medical director of third-party CRO (QPS Holdings, LLC) which is conducting the clinical trial. The funder Foresee Pharmaceuticals played no role in such determination. These included blurred vision, left hip fracture, and myocardial infarction.

### Patient-reported outcomes

The average change in degree of bone pain from baseline was 0.07 ± 1.666 on Day 168 and 0.24 ± 1.988 on Day 336. The average change in degree of urinary pain from baseline was − 0.07 ± 0.847 on Day 168 and 0.09 ± 1.280 on Day 336. None of these differences was statistically significant.

Of the 137 subjects, approximately 68.6% (94/137) felt satisfied [answered quality of life (QoL) questionnaire with 0—delighted, 1—pleased, or 2—mostly satisfied] with their lives at current condition at baseline. The respective figures on Day 168 and Day 336 were 69.7% (90/129) and 65.9% (87/132), indicating that the administration of LMIS 50 mg did not appear to cause additional discomfort in subjects during the study.

### Pharmacokinetics

The PK profiles of LMIS 50 mg exhibited two phases. After dosing, an initial rapid increase of serum leuprolide concentration was observed, followed by a rapid decline over the first 3 days post-dose. The initial rapid increase was characterized by mean high serum concentrations (> 90 ng/mL), and the mean serum leuprolide levels maintained relatively constant over the majority of each 6-month dosing interval. Leuprolide appeared to be released continuously by the third day after dosing with steady serum concentrations (“plateau” phase) through the 6-month dosing interval (mean concentration: 0.370–2.97 ng/mL). The serum leuprolide concentrations and the associated pharmacokinetics following the first and second doses of LMIS 50 mg were similar, which suggested lack of significant accumulation with repeated dosing at 6-month intervals. The PK parameters following first and second dose administrations in Part I and Part II studies are summarized in Table 4.

**Table 4:** Summary of serum PK parameters of leuprolide after LMIS 50 mg subcutaneous injections to subjects with prostate carcinoma

PK parameters	Part I						Part II					
	First dose			Second dose			First dose			Second dose		
	*N*	Mean	SD	*N*	Mean	SD	*N*	Mean	SD	*N*	Mean	SD
*C*_max_ (ng/mL)	31	94.5	53.7	29	99.0	73.0	94	99.7	65.6	97	93.7	60.8
*T*_max_ (h)^a^	31	3.23 (1.17, 7.90)		29	2.08 (1.17, 8.00)		94	3.67 (2.83, 24.00)		97	3.78 (2.87, 5.17)	
*C*_wk4_ (ng/mL)	31	1.04	0.863	29	1.64	0.983	94	1.47	2.57	96^c^	2.40	4.05
*C*_mon6_ (ng/mL)	29^a^	0.497	0.610	29	0.511	0.488	92^b^	0.370	0.313	94^c^	0.410	0.538
AUC_0–4wks_ (day ng/mL)	31	91.6	47.9	29	125	57.3	94	103	62.4	96^c^	131	91.4
AUC_0–6mon_ (day ng/mL)	29^a^	224	87.3	29	268	88.1	92^b^	219	108	94^d^	250	160
*C*_avg(0–6mon)_ (ng/mL)	29^a^	1.34	0.519	29	1.59	0.525	92^b^	1.31	0.643	94^d^	1.49	0.950

*T*_*max*_ median (Min, Max), *N* number of subjects, *SD* standard deviation

^A^two subjects PK parameters were not reportable

^b^Not reportable for 2 subjects

^C^not reportable for 1 subject

^d^Not reportable for 3 subjects

### Limitations

This study is an open-label, single-arm study design and no direct safety and efficacy comparisons to reference drug can be done. The study duration was 12 months and two injections with 6 months apart were given in these prostate cancer subjects. 30 subjects were given additional two injections in an extension safety study (total observation 24 months) that showed no evidence of safety and tolerability concerns. Thus, there is no long-term safety or efficacy data beyond 24 months available at this point.

## Discussion

The phase 3 trial results show that the first injection of LMIS 50 mg was effective in achieving testosterone suppression to castrate levels (≤ 50 ng/dL) by Day 28 in 98% of subjects in this study, and that two doses of LMIS 50 mg (6 months apart) successfully suppressed the serum testosterone to castrate levels in 97% of total subjects at the end of the study (approximately 1 year). Using a more stringent criterion for castration [16], 95.9% of the patients had serum testosterone level < 20 ng/dL at the end of the study.

Although two subjects exhibited the post-suppression excursion of serum testosterone to > 50 ng/dL after achieving the castration level of testosterone on Day 28, which was presumably due to the acute-on-chronic surge following the second administration of LMIS 50 mg, the percentage of subjects exhibiting post-suppression elevation of serum testosterone to > 50 ng/dL was 0% in both ITT and PP populations at the end of the study.

Recognizing the caveats of cross-study comparisons [17], LMIS 50 mg demonstrated similar efficacy to other LH–RH agonists that are administered once every 6 months for reducing and maintaining the serum testosterone to castration levels during the study period (Table 5). Overall, the incidence of drug-related AEs after the administration of LMIS 50 mg in the present study was also comparable to the incidence of drug-related AEs for similarly approved LH–RH analogs [8, 18], typically hot flush being the most frequent adverse event. Furthermore, the administration did not cause any additional unexpected adverse events for this class of drug administered for this patient population.

**Table 5:** Cross-study comparison of efficacy end points between LMIS 50 mg and two approved 6-month depot formulations of leuprolide acetate (8, 18)

Efficacy and safety assessment	LMIS 50 mg	6-month subcutaneous depot of leuprolide acetate 45 mg (8)	6-month intramuscular depot of leuprolide acetate 45 mg (18)
Total number of enrolled subjects	137	111	151
Number of subjects in PP population	124	109	148
Mean testosterone level at baseline (ng/dL) (PP population)	475.2	367.7	435
Mean testosterone level on Day 2 (ng/dL) (PP population)	695.7^a^	588.6	608
Mean testosterone level on Day 28 (ng/dL) (PP population)	17.4	16.7	< 16
Mean testosterone level on Day 336 (ng/dL) (PP population)	9.95	12.6	NA
Percentage of subjects reached serum testosterone castration level (< 50 ng/dL) on Day 28 (PP population)	99.2 (123/124)	99.1 (108/109)	99.3 (147/148)
Percentage of total subjects with failed suppression of serum testosterone to castrate levels (any testosterone value > 50 ng/dL, including breakthrough and acute-on-chronic) (PP population)	2.4 (3/124)	1.8 (2/109)	6.08 (9/148)
Percentage of subjects reached serum testosterone castrate level (< 50 ng/dL) from Day 28 to Day 336 (PP population)	97.6 (121/124)	99 (102/103)	93.4 (138/148)

^a^8 h post dosing

With regard to PK, LMIS 50 mg resulted in a multi-phasic leuprolide concentration versus time profiles characterized by a distinctive burst phase and a plateau phase. The initial acute increase of leuprolide mesylate concentration, followed by the rapid decline to a steady-state level, was similar to the release pattern seen with the other leuprolide depot formulations. After an initial burst phase characterized by mean high serum leuprolide concentrations (> 90 ng/mL), mean serum leuprolide levels maintained relatively constant over the majority of each 6-month dosing interval. The serum leuprolide concentrations and the associated pharmacokinetics following the first and second doses of LMIS 50 mg suggested lack of significant accumulation with repeated dosing at 6-month intervals.

LMIS 50 mg was developed as a pre-filled, ready-to-use syringe that does not require reconstitution prior to subcutaneous injection. Adequate reconstitution of LH–RH analogs is essential for the administration of products that do require premixing, to safeguard optimal and effective treatment of prostate cancer patients. Since preparation errors have been reported which have been associated with lack of drug efficacy, including increase of testosterone levels above the castrate level and/or increase of PSA levels [15], eliminating this administrative step could, thus, decrease a potential source of iatrogenic therapeutic inefficiency for the treatment of prostate cancer patients.

In conclusion, LMIS 50 mg achieved the therapeutic goal of suppressing the serum testosterone to the castration level in 98% subjects on Day 28 and the suppression rate was maintained in 97% of the subjects throughout end of this study. Two separate doses of LMIS 50 mg administered 6 months apart over a 12-month interval demonstrated similar safety and PK profile when compared to marketed products for the treatment of advanced prostate cancer patients, with the associated advantage of eliminating the need for manual reconstitution.
